# Patient involvement in medication safety in hospital: an exploratory study

**DOI:** 10.1007/s11096-014-9951-8

**Published:** 2014-04-29

**Authors:** Soomal Mohsin-Shaikh, Sara Garfield, Bryony Dean Franklin

**Affiliations:** 1 The Centre for Medication Safety and Service Quality, Imperial College Healthcare NHS Trust, Charing Cross Hospital, Fulham Palace Road, London, W6 8RF UK; 2 UCL School of Pharmacy, 29-39 Brunswick Square, London, WC1N 1AX UK

**Keywords:** Hospital, Medication safety, Patient participation, Shared decision making, United Kingdom

## Abstract

*Background* Medication errors are common in hospital inpatients. While many interventions have been proposed to address these problems, few have been shown to have significant benefits. A complementary approach is to facilitate greater involvement of patients with their inpatient medication. However, there is relatively little research in this area and it is not known which interventions lead to improved healthcare outcomes. Work is therefore needed to investigate the roles that healthcare professionals and patients believe are appropriate for hospital inpatients to take relating to safety. *Objective* To explore the extent to which hospital inpatients reported that they engaged with medication safety-related behaviours, the extent to which they would like to, and the extent to which healthcare professionals reported that they would support such engagement. *Setting* An NHS hospital Trust in West London. *Methods* 100 Patients and healthcare professionals were recruited on ten wards within the Trust and invited to complete quantitative questionnaires. Data were analysed descriptively and exploratory comparisons made between different groups of respondents. *Main outcome measures* inpatient medication safety involvement scale and control preference scale for patient involvement in decision making. *Results* 100 patients (98 % response rate) and 104 healthcare professionals (59 % response rate) were recruited. The majority of patients and healthcare professionals were supportive of hospital inpatients being involved with their medication. However there was a significant gap between desire for patient involvement and what patients reported having experienced. Female patients and those under 65 wanted a significantly higher level of involvement than males and over 65s. Few associations were found between healthcare professionals’ reported support for involvement and their profession or gender. However, pharmacists and nurses were significantly more likely to report supporting patients asking questions about their medicines and self administering their own medicines than doctors. *Conclusion* Healthcare professionals and patients desire a higher level of patient involvement with their medication while in hospital than patients currently report. Interventions need to be developed to bridge the gap between desired and actual patient involvement.

**Electronic supplementary material:**

The online version of this article (doi:10.1007/s11096-014-9951-8) contains supplementary material, which is available to authorized users.

## Impact of findings on practice

- The gap between patients’ preferred and experienced involvement with inpatient medication needs to be addressed.
- Healthcare professionals would support patients in being involved with their medication while in hospital, but they need to find ways to encourage this in practice.

## Introduction

The UK, USA and the World Health Organization [1–4] have identified that priority should be given to improved patient safety in healthcare. Medication error has been shown to be one of the most frequent forms of medical error and is associated with significant harm [5]. Medication errors are common in hospital inpatients. UK research suggests that prescribing errors occur in up to 15 % of inpatient medication orders, commonly involving omission of patients’ usual medication on admission, and 9 % of medications prescribed at discharge [6]. A recent meta-analysis reports medication administration errors in 5.6 % of non-intravenous doses and 35 % of intravenous doses administered in the UK [7]. Although many of these do not result in patient harm, others have more serious consequences [8], and even errors which do not cause harm can seriously affect the patient’s confidence in their healthcare.

While many interventions have been proposed to address these problems, few have been shown to have significant benefits [9]. A complementary approach, not yet widely studied, is to facilitate greater involvement of patients with their inpatient medication. In particular, patients (and their carers) are likely to know a great deal about medication that they have been using prior to admission. Patients are therefore a potentially important (and often the final) defence against errors relating to their medication.

Patient safety activities relating to inpatient medication include, but are not limited to, patients viewing their inpatient medication records, prompting staff to avoid dose omissions, providing information to aid handover between shifts and professional groups, and raising queries with prescribers, pharmacists or nursing staff. For example, in one Swiss study, oncology patients detected errors such as dose omissions [10]. Locally, observation of medication administration rounds has confirmed that patients do query their medication with ward staff, and sometimes prevent potential medication errors [11]. However, our experience also suggests that patients are often unsure of the medication they are prescribed as an inpatient, preventing effective engagement with their treatment and thus a more active role in medication safety. In particular, we have observed considerable confusion among both patients and hospital staff regarding whether or not hospital inpatients are ‘permitted’ to look at their hospital medication records where their current medication is prescribed and recorded.

Research has shown that patients are more willing to participate in patient safety if encouraged to do so by healthcare professionals [12–14]. However, there is relatively little research in this area. A patient partnership intervention has not shown a significant difference in adverse drug effects between intervention and control groups [15] and it is not known which interventions lead to improved healthcare outcomes [16]. Further work is therefore needed to investigate the roles that healthcare professionals and patients believe are appropriate for hospital inpatients to take relating to safety [17].

### Aims of the study

In this paper we report the findings of a service evaluation which aimed to explore the extent to which hospital inpatients reported that they engaged with medication safety-related behaviours, the extent to which they stated they would like to, and the extent to which healthcare professionals reported that they would support such engagement.

#### Objectives

- To explore patients’ views on being involved in different aspects of their medication while in hospital.
- To explore healthcare professionals’ views on patients being involved in different aspects of their medication while in hospital.
- To explore whether views on patient involvement might differ between different healthcare professions, age groups and gender.
- To explore whether desired and reported patient involvement differ among patient genders and age groups.
- To identify any mismatches concerning views on involvement between patients and healthcare professionals.
- To identify any mismatches between the involvement patients would like and their experienced involvement.

## Methods

This exploratory survey was exempt from ethical review and was approved as a service evaluation by the Quality and Safety Committee of the study Trust.

### Setting

The study took place in three hospitals of an NHS Trust in West London in April and May 2013. There were a total of 82 wards in the Trust, each typically comprising about 20–25 beds. The Trust operated typical UK systems for prescribing, dispensing and administration of medication. Prescribing was paper-based, using pre-formatted drug-charts on which nurses also recorded medication administration. Commonly used medication was kept as ward stock with other medication dispensed for individual patients. Patients were also encouraged to bring their own medication into hospital. Procedures were in place to allow patients to self-administer medication where appropriate. Pharmacists visited each ward on weekdays to check that medication orders were clear, legal and clinically appropriate for each patient and to initiate the supply of any medication needed.

### Participants

We excluded wards thought likely to have a very high proportion of unwell patients, as well as private wards. Convenience sampling [18] was then used to recruit ten wards from those remaining and then to identify patients and healthcare professionals on the participating wards. We studied a wide range of wards: gastroenterology, infectious diseases, medical admissions, gastrointestinal surgery and urology, trauma and orthopedics, elderly care rheumatology and endocrine, urogynaecology, post-natal and two stroke wards.

Healthcare professionals on participating wards assisted with the identification of patients likely to meet our inclusion criteria. Patients not speaking English, patients judged by the healthcare professionals to be too unwell or too cognitively impaired to participate and patients under 18 were excluded. The researcher (SMS) then approached potentially suitable patients, provided them with a verbal explanation of the study and offered a patient information leaflet. Patients were able to take as much time as they required to make a decision on whether to participate. Completion of the questionnaire was taken as consent.

When visiting a ward, the researcher (SMS) approached healthcare professionals on the ward at that time. All ward pharmacists within the Trust were approached by email. Respondents were given a brief explanation about the study and offered further written information if required. Completion of the questionnaire was taken as consent.

The target sample size was 100 patients and 100 healthcare professionals to allow for exploratory comparisons between different genders, age groups and healthcare professions.

### Instruments

We used separate quantitative Questionnaires for healthcare professionals and patients (available online as supplementary material). These both comprised two scales; the first was developed for the present study, referred to as the inpatient medication safety involvement scale (IMSIS). The patient version comprised eight exploratory questions about views on patient participation in their medication in general while in hospital and medication safety in particular. The healthcare professional version had five questions as we asked for their views only, and not their experiences. We developed the IMSIS scale with reference to the literature [19–21] and in line with our research objectives. The second was the three item control preference scale [22], a validated instrument for measuring preferences for involvement in healthcare decision making, as adapted by Garfield et al. [23] to apply specifically to medication. The questionnaire also included questions on gender, age, and (for healthcare professionals) profession. The researcher piloted the questionnaire on the participating wards with nine healthcare professionals and five patients, and assessed responses for face and content validity and acceptability. Following piloting, a question on whether or not pharmacists were qualified independent prescribers was amended very slightly to make it clearer. As no other amendments were made, the pilot Questionnaires completed were included in the main study.

### Data collection

The patients and healthcare professionals were presented with the questionnaire on the ward by the researcher who then collected the completed questionnaires. Respondents were able to take as much time as they wanted to complete the questionnaire. If a patient was unable to complete the questionnaire themselves, the researcher offered to read the questionnaire and complete it on their behalf. Questionnaires were also emailed to all the pharmacists working at the Trust and emailed back to the researcher; one reminder was sent 11 days later.

### Data entry and analysis

Data were entered onto an SPSS (version 21) database which was then cleaned. Descriptive quantitative data were generated for all variables. We used Kreskas Wallis and Mann–Whitney U tests to investigate whether there were significant differences between the views of different groups of healthcare professionals and between different genders and age groups of patients and healthcare professionals. Wilcoxon tests were used to test whether there were significant differences between patients’ preferred involvement and the involvement they experienced. We used Cronbach’s alpha to determine the internal reliability of both our new instrument on participation in medication safety (IMSIS) and the control preference scale [22, 23]. This provided information about whether or not it would be appropriate to combine the individual items into a scale and use summed scores for each of the two scales in further analysis. We calculated Cronbach’s alpha separately for patients and healthcare professionals’ data but did not sub-divide healthcare professionals by profession as the numbers would have been too small for meaningful analysis. Where appropriate, we used Spearman’s bivariate correlation to determine the strength of association between the two scales.

While this was primarily a quantitative study, any comments spontaneously added by respondents to the questionnaire or stated verbally to the researcher that informed the research objectives were recorded and analysed descriptively.

## Results

### Response rates

One hundred patients (98 % response rate), 24 doctors (80 % response rate), 30 pharmacists (31 % response rate) and 50 nurses (100 % response rate) took part. Demographic data for both patients and healthcare professionals are presented in Table 1. The doctors who did not participate reported that they were too busy. One patient who did not participate reported that s/he was too tired and the other that s/he did not want to be involved with the study.

**Table 1:** Respondent characteristics

Total sample size	Patients	Healthcare Professionals
	100	104
Gender		
Male	34	28
Female	66	76
Age (n = 99^a^)		
≤65	56	–
>65	44	–
Type of Healthcare Professional		
Doctor	–	24
Pharmacist	–	30
Nurse	–	50

^a^Age was missing for one patient

### Patients’ and healthcare professionals’ views on inpatient involvement with medication, medication safety and prescribing decisions

**Fig. 1:**
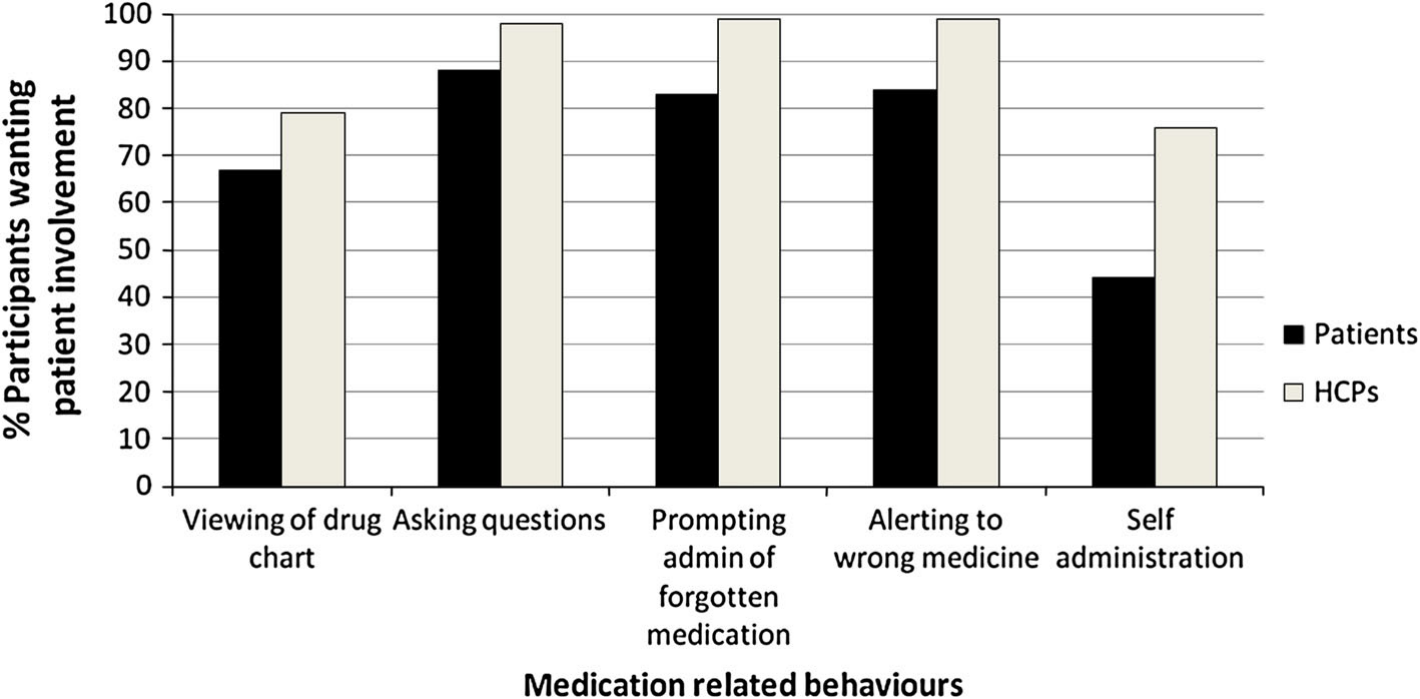
Preferences for inpatient involvement with medication according to the IMSIS scale. HCPs: health care professionals

The majority of patients and healthcare professionals were supportive of hospital inpatients being involved with their medication. Table 2 shows the level of involvement that patients both wished to have and actually reported having with different aspects of their medication, according to the IMSIS scale. Wilcoxon tests demonstrated a significant difference between the level of desired and experienced involvement, with patients wanting more involvement than they had actually experienced (Table 3). Table 4 shows the level of support that healthcare professionals reported that they would have for patient involvement. Descriptive exploration of the results for each individual item in IMSIS suggests a trend towards healthcare professionals being more likely to say that they would support patient involvement than patients wanting such involvement. This is illustrated further in Fig. 1.

**Table 2:** Patients’ preferences and actual involvement with medication and medication safety in hospital, using the IMSIS scale (n = 100)

	‘I have looked at my medication admin record (drug chart) while in hospital’ (%)	I would like to look at my medication admin record (drug chart) while in hospital (%)	‘I have asked questions about my medicines while in hospital’ (%)	‘I would like to ask questions about my medicines while in hospital’ (%)	‘I have kept and administered my medicines while in hospital’ (%)	‘I would like to keep and administer my own medicines while in hospital’ (%)	‘I would check with a healthcare professional if I thought I might be being given the wrong medicine’ (%)	‘I would check with a healthcare professional if I thought one or more of my medicines have been forgotten’ (%)
Strongly agree	8	15	21	25	8	13	43	34
Agree	21	52	55	63	12	31	41	49
Uncertain	5	7	0	0	9	13	3	3
Disagree	35	23	17	10	41	39	12	14
Strongly disagree	31	3	7	2	30	4	1	0

**Table 3:** Differences between desired and experienced patient involvement in different aspects of medication safety while in hospital, according to the IMSIS scale

Statements	Test	Significance
I have looked at my drug chart while in hospital versus I would like to look at my drug chart while in hospital	Related samples- Wilcoxon Signed Rank Test	*p* < 0.001
I have asked questions about my medicines while in hospital versus I would like to ask questions about my medicines while in hospital		
I have kept and administered my medicines while in hospital versus I would like to keep and administer my own medicines while in hospital		

Items measuring desired involvement without a matching item for experienced involvement not included

**Table 4:** Healthcare professionals’ support for patient involvement with medication in hospital, according to the IMSIS scale (n = 104)

	‘I would support patients looking at their medication administration record (drug chart) while in hospital’ (%)	‘I would support patients asking questions about their medicines while in hospital’ (%)	‘I would support patients in checking with a healthcare professional if they thought one or more of their medicines had not been prescribed’ (%)	‘I would support patients in checking with a healthcare professional if they thought one or more of their medicines had been prescribed but not administered’ (%)	‘I would support patients in checking with a healthcare professional if s/he thought they might have been given the wrong medicine’ (%)	‘I would support patients in self administering their own medicines while in hospital’ (%, one)
Strongly agree	35	77	80	80	83	41
Agree	44	21	19	19	16	35
Uncertain	12	2	0	0	0	9
Disagree	5	0	1	1	1	9
Strongly disagree	4	0	0	0	0	6
Missing	0	0	0	0	0	1

**Fig. 2:**
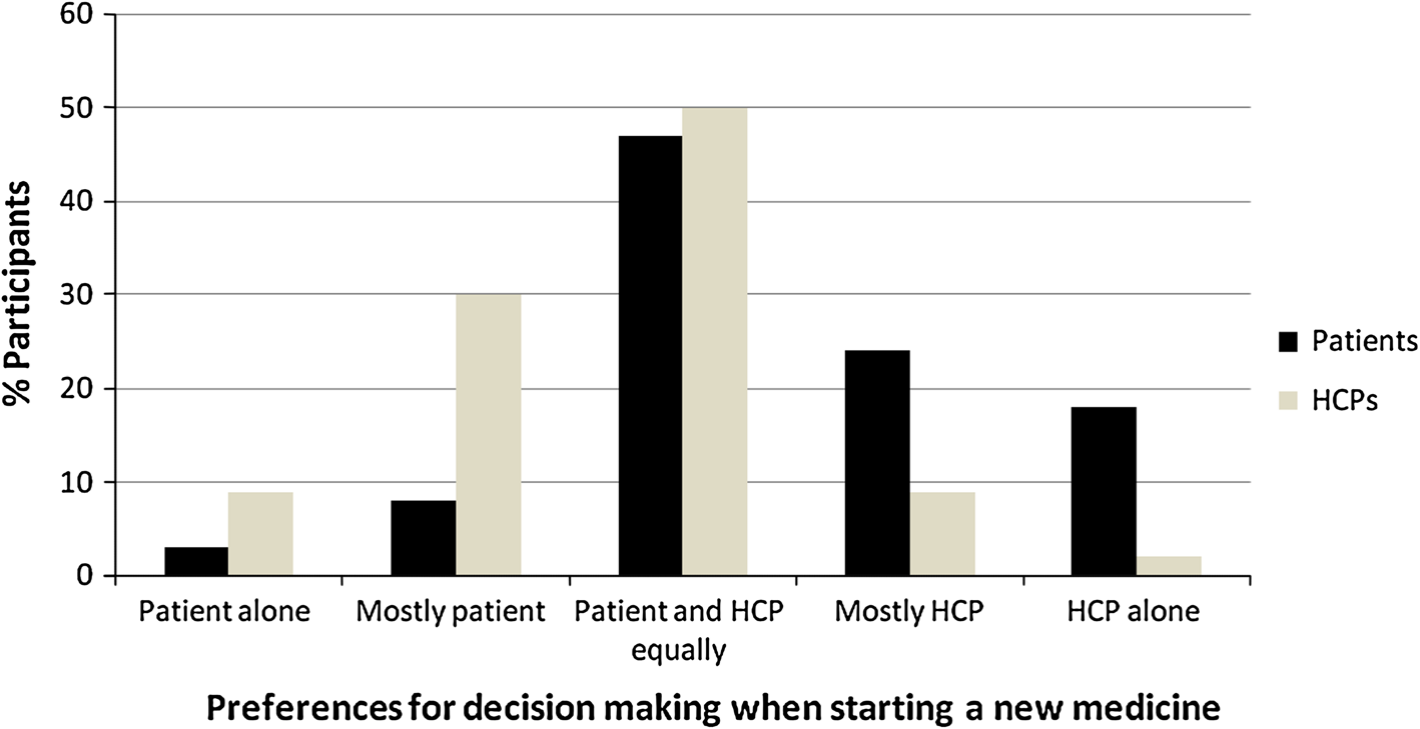
Preferences for decision making when starting a new medicine using the adapted control preference scale. HCPs: health care professionals

Table 5 and Fig. 2 shows patients’ and healthcare professionals’ preferences regarding who should make decisions about their medication, according to the control preference scale. Once again, there appeared to be a trend towards healthcare professionals reporting a preference for greater patient involvement in decision making than patients had themselves.

**Table 5:** Patients’ and healthcare professionals’ preferences for involvement in decisions regarding their medication (adapted Control Preference Scale)

	Starting a new medicine		Changing the dose of a medicine that the patient is already taking		Stopping a medicine	
	Patient (n = 100)	Healthcare professionals (n = 104)	Patient (n = 100)	Healthcare professionals (n = 104)	Patient (n = 100)	Healthcare professionals (n = 104)
Patient alone	3	9	2	7	2	6
Mostly patient	8	30	11	16	10	13
Doctor (or other healthcare professional) and patient equally	47	50	45	52	46	45
Mostly Doctor (or other healthcare professional)	24	9	26	20	30	27
Doctor (or other healthcare professional) alone	18	2	16	5	12	9
Total (%)	100	100	100	100	100	100

### Internal reliability of instruments used

For both scales (IMSIS and the control preference scale), patients’ data showed good internal consistency (Cronbach’s alpha >0.7) suggesting it was appropriate to sum the individual items within the scale concerned to give overall scores for each scale for the remaining analysis (Table 6). However, the healthcare professionals’ data did not show the same level of internal consistency for either scale (Cronbach’s alpha <0.7); therefore in the remainder of the analysis, we consider healthcare professionals’ responses to each individual item rather than considering an overall score for each scale.

**Table 6:** Internal reliability of instruments used

Scale	Patients	Healthcare professionals
Cronbach’s Alpha		
IMSIS	0.733	0.574
Adapted control preference scale	0.857	0.665

### Association between preference for general involvement with inpatient medication and involvement in decision making

For patients, there was a weak correlation between IMSIS scores and those for the control preference scale (Spearman’s correlation coefficient = 0.41, *p* < 0.001). It was not appropriate to carry out this correlation test for healthcare professionals as Cronbach’s alpha suggested internal reliability for each scale to be low.

### Associations between patient involvement and age/gender

Mann–Whitney U tests demonstrated that female patients (*p* = 0.003) and patients 65 and under (*p* = 0.002) had significantly higher scores for overall desired and experienced involvement with their medication in hospital than males and those over 65 respectively (Table 7). Females (*p* < 0.001) and those 65 and under (*p* < 0.001) were also more likely to want to be involved in decision making regarding medicines (Table 8).

**Table 7:** Comparisons between gender and age groups for patients’ IMSIS scale (Mann–Whitney U tests)

Total sample (n = 100)	Mean	Standard deviation	*p* value*
	20.89	5.308	
Gender			
Male (n = 34)	23.21	4.663	0.003
Female (n = 66)	19.70	5.256	
Age			
≤65 (n = 56)	19.52	5.350	0.002
65< (n = 43)	22.84	4.629	

* Asymptotic significance values are displayed. The significance level is 0.05

The lower the score, the greater the involvement

**Table 8:** Comparisons between gender and age groups for patients’ responses to the adapted Control Preference Scale (Mann–Whitney U tests)

Total sample (n = 100)	Mean	Standard deviation	*p* value*
	10.29	2.500	
*Overall score for involvement with decisions*			
Gender			
Male (n = 34)	11.44	2.048	<0.001
Female (n = 66)	9.70	2.517	
Age			
≤65 (n = 56)	9.50	2.296	<0.001
65< (n = 43)	11.33	2.427	

### Associations between healthcare professional support for patient involvement and gender/healthcare professional type

When individual items were tested, there were few associations between healthcare professional support for patient involvement with gender or healthcare professional type. However, Kruskal–Wallis (Table 9) and Mann–Whitney U tests showed that both pharmacists and nurses were significantly more likely to report that they would support patients asking questions about their medicines and self administering their own medicines than doctors (*p* < 0.001).

**Table 9:** Comparisons among healthcare professional groups in terms of their support for patients asking questions about their medicines and self-administering (Kruskal–Wallis tests)

Statement	Mean ranks		Chi square statistic	Degrees of freedom	*p* value
I would support patients asking questions about their medicines while in hospital’	Doctor	69.13	20.479	2	<0.001
	Pharmacist	42.20			
	Nurse	50.70			
‘I would support patients in self administering their own medicines while in hospital’	Doctor	70.98	16.264	2	<0.001
	Pharmacist	40.45			
	Nurse	49.78			

### Barriers to patient involvement

Comments spontaneously added by respondents to the questionnaire or stated verbally to the researcher provided preliminary data regarding some of the barriers to patients being more involved with their medicines.

When patients were asked (1) if they would check with a healthcare professional if they thought one or more of their medicines may have been forgotten or (2) they might be being given the wrong medicine, two expressed the opinion that they trusted the healthcare professionals’ expertise and would not challenge them.

One patient reported that patients’ medicines may be changed or stopped while in hospital and that they may not have enough knowledge to self-administer their own medicines.

Three healthcare professionals cited patients’ lack of cognitive ability and one cited patients’ limited English as barriers to patient involvement. One nurse expressed the view that he did not have enough information about self administration of medicines to say whether or not he would support this. Some doctors reported that they would not like to be personally involved with self administration as they had other demands on their time; they would prefer nurses to take the lead on this.

## Discussion

Both patients and healthcare professionals reported that they supported patient involvement in medication and medication safety. This supports the findings of Davis et al. [12, 17] when exploring patients’ and healthcare professionals’ views on patients general involvement in healthcare and safety and those of Schwappach and Wernli [14] when investigating chemotherapy patients’ engagement in medical error prevention. However, Davis et al. [12] found that patients reported lower willingness to notify doctors of problems or errors than to ask factual questions, but a higher willingness to notify nurses of errors than to ask factual questions. Other studies have shown that patients are more willing to participate in behaviours that were less challenging [14, 24]. In our study, a similar proportion of patients wanted to ask questions about their medicines as said they would challenge a healthcare professional if they thought an error was being made. The reason for the difference in findings may be due to the fact that our study asked about healthcare professionals in general, rather than asking separately about doctors, nurses and pharmacists.

Female patients and those 65 and under wanted significantly more involvement in medication and medication safety than males and those over 65. Findings from previous studies regarding the association between age and gender and preferences for involvement in healthcare safety have been inconclusive [21]. However, the finding that younger patients have higher preference for involvement in decisions about medicines is consistent with other research [23, 25]. Previous research has also shown that younger patients are more likely to have a preference towards self administration of medicines in hospital [23]. Previous findings concerning gender and preference for involvement in decision making have been mixed [23, 25]. However, all studies that have identified an association with gender have concluded that women are the more likely to prefer a more active role [25]. In addition, female patients have been shown to be more likely to show a preference towards self administration of medication in hospitals [26].

Few associations were found between healthcare professional type and gender, and their preference for involvement. However, pharmacists and nurses were significantly more likely to support patients asking questions and self administering their medicines than doctors. Davis et al. [17] found that nurses were more likely to support patient involvement in safety than doctors.

There was a descriptive trend towards healthcare professionals reporting being more supportive of patient involvement with medication than patients in the individual items of both the IMSIS and control preference scales. Davis et al. [17] found that doctors were more likely to support patient involvement in safety as healthcare professionals than they would involve themselves as patients, although this effect was not observed for nurses.

Healthcare professionals showed less variation than patients in which specific patient involvement behaviours they strongly supported (Tables 2, 4). Whilst both the IMISS and control preference scale had good internal reliability for patients, neither scale showed good internal reliability for healthcare professionals, suggesting that healthcare professionals’ beliefs may not form one scale. This is the first study we are aware of where the control preference scale had been used for healthcare professionals. The results suggest that it may not be appropriate to group the items as one scale in analysis.

### Strengths and limitations

Our study reported important exploratory findings regarding patients’ and healthcare professionals’ views on patient involvement in medication safety while in hospital. Unlike many studies in this field we included pharmacists as well as doctors and nurses. Limitations included that it was carried out in one trust and convenience sampling was used. However, patients were recruited from a variety of ward types and the response rates were mostly very high. The response rate for pharmacists was lower as fewer were present on the wards at the time of our researcher’s visits and they therefore generally had to be recruited via email rather than in person. However, they were not likely to have represented a dramatically different range of specialties to healthcare professionals recruited on the wards because most pharmacists in the Trust each provide services to a wide range of wards. For the same reason, we do not have data on the total number of nurses and doctors working on the study wards and are therefore unable to calculate the proportion of these who completed our questionnaire. The views of patients who were too unwell to participate or did not speak English were not represented. Numbers for each healthcare professional group are relatively low, precluding anything other than exploratory analysis. Another limitation was that some doctors appeared to interpret supporting patients in self administration of medicines as personally setting up patients for self administration rather than being generally in support of the principle. This may have made their support appear lower than it was. None of the doctors interpreted the questionnaire in that way during our piloting so this issue was not identified at that stage.

### Implications for practice

The gap between patients’ preferred and experienced involvement with inpatient medication needs to be addressed. Clinical pharmacists are medication experts with direct contact with patients and thus potentially have an important role to play in closing the gap. A high proportion of healthcare professionals state that they would support patients in being involved with their medication while in hospital, but they need to find ways to encourage this in practice. Davis et al. [12] have shown that patients are more likely to participate in safety behaviours if encouraged to do so by healthcare professionals. More research is needed to understand the barriers to involving patients with their medication while in hospital and interventions should be developed to facilitate involvement. As a follow up to this study, we are planning to conduct an in depth qualitative study to address these issues.

## Conclusion

The majority of patients and healthcare professionals were supportive of hospital inpatients being involved with their medication. However there was a significant gap between this desire for patient involvement and what was reported to be experienced by patients in practice. Female patients and those under 65 wanted a significantly higher level of involvement with their medication than males and those over 65. This finding needs to be taken into consideration when developing interventions. Few associations were found between healthcare professional support for involvement and their profession and gender. However, pharmacists and nurses were significantly more likely to support patients asking questions about their medicines and self administering their own medicines than doctors.

## Electronic supplementary material

Below is the link to the electronic supplementary material.

Supplementary material 1 (RTF 172 kb)
