# Supplementary material for: Patient involvement in medication safety in hospital: an exploratory study
Source: Int J Clin Pharm. 2014 Apr 29;36(3):657–66. doi: 10.1007/s11096-014-9951-8 (PMC4019827; doi:10.1007/s11096-014-9951-8)
Supplement: Supplementary file 1 — Supplementary material 1 (RTF 172 kb) [file 11096_2014_9951_MOESM1_ESM.rtf]

Patient Questionnaire
Date of Birth:....................................................
Gender:  	Male  	Female 
Ward:.................................................. 
1) Here are a series of statements about your medicines. For each statement, please tick the appropriate box to indicate the extent to which you agree or disagree with it: 
	Strongly agree
	Agree	Uncertain	Disagree	Strongly disagree	
I have looked at my medication administration record (drug chart) while in hospital 
	
					
I would like to look at my medication administration record (drug chart) while in hospital 
						
I have asked questions about my medicines while in hospital 
						
I would like to ask questions about my medicines while in hospital 
						
I would check with a healthcare professional if I thought one or more of my medicines may have been forgotten 
	
	
	
	
	
	
I would check with a healthcare professional if I thought I might be being given the wrong medicine 
	
	
	
	
	
	
I have kept and administered my medicines while in hospital 
						
I would like to keep and administer my own medicines while in hospital 
						


Please continue over to page two

2) When thinking about starting a new medicine, if the doctor (or other healthcare professional) has told you about its benefits and risks, who should finally decide about your treatment? Please tick ONE of the below: 
You alone 						     
Mostly you 						     
Doctor (or other healthcare professional) and you equally   

Mostly the doctor (or other healthcare professional)             

Doctor (or other healthcare professional) alone                      


3) When thinking about changing the amount of a medicine that you are already taking, if the doctor (or other healthcare professional) has told you about the benefits and risks of doing so, who should finally decide about your treatment? Please tick ONE of the below: 

You alone 						
Mostly you 						
Doctor (or other healthcare professional) 		
and you equally

Mostly the doctor (or other healthcare professional)	

Doctor (or other healthcare professional) alone 		

4) When thinking about stopping a medication, assuming that the doctor (or other healthcare professional) has told you about the benefits and risks of doing so, who should finally decide about your treatment? Please tick ONE of the below: 

You alone 						
Mostly you 						
Doctor (or other healthcare professional 		
and you equally

Mostly the doctor (or other healthcare professional)	

Doctor (or other healthcare professional) alone 		
Thank you for your participation. Please check you have completed all the sections
Healthcare Professional Questionnaire
Please tick one of the following:      Doctor 	               Pharmacist  		  Nurse 
Gender:  	Male  	Female 
Date of Birth:....................................................
Pharmacists only, please tick one of the following:	
Are you qualified as an independent prescriber? 	Yes   	No    
1) Here are a series of statements about inpatient involvement with their medication in hospital. For each statement, please tick the appropriate box to indicate the extent to which you agree or disagree with it: 
	Strongly agree 
	Agree 	Uncertain	Disagree	Strongly disagree	
I would support patients looking at their medication administration record (drug chart) while in hospital
						
I would support patients asking questions about their medicines while in hospital 
						
I would support patients in checking with a healthcare professional if they thought one or more of their medicines had not been prescribed 
	
	
	
	
	
	
I would support patients in checking with a healthcare professional if they thought one or more of their medicines had been prescribed but not administered 
 	
	
	
	
	
	
I would support patients in checking with a healthcare professional if s/he thought they might have been given the wrong medicine 
	
	
	
	
	
	
I would support patients in self administering their own medicines while in hospital 						


2) When thinking about starting a new medicine, if the doctor (or other healthcare professional) has told the patient about its benefits and risks, who should finally decide about the treatment? Please tick ONE of the below: 

Patient alone 						
Mostly the patient 					
Doctor (or other healthcare professional)		 
and patient equally					

Mostly the doctor (or other healthcare professional)	

Doctor (or other healthcare professional) alone	               

3) When thinking about changing the dose of a medicine that the patient is already taking, if the doctor (or another healthcare professional) has told the patient about the benefits and risks of doing so, who should finally decide about the treatment? Please tick ONE of the below: 

Patient alone 						
Mostly the patient					
Doctor (or other healthcare professional)		
and patient equally

Mostly the doctor (or other healthcare professional)	

Doctor (or other healthcare professional) alone 		

4) When thinking about stopping a medication, assuming that the doctor (or other healthcare professional) has told the patient about the benefits and risks of doing so, who should finally decide about the treatment? Please tick ONE of the below: 

Patient alone 						
Mostly the patient  					
Doctor (or other healthcare professional) 			
and patient equally

Mostly the doctor (or other healthcare professional)	

Doctor (or other healthcare professional) alone 		
